# TWO SIDES OF THE SAME COIN: OPIOIDS AND CANNABIS FOR PAIN MANAGEMENT IN AFRICAN AMERICAN OLDER ADULTS

**DOI:** 10.1093/geroni/igad104.2805

**Published:** 2023-12-21

**Authors:** Simone Jackson, Staja Booker

**Affiliations:** University of Florida College of Nursing, Gainesville, Florida, United States; University of Florida, Gainesville, Florida, United States

## Abstract

Approximately 50 million U.S. adults live with chronic pain. Example treatments for chronic pain include opioids and the re-emerging cannabinoid pain management (CPM). However, older African Americans (AAs) are more likely to use pain treatments that are considered safe, affordable, and socially acceptable. We examined the perception of opioid and cannabis use for pain management in AA older adults by conducting: (1) secondary data analyses from two studies to quantify patterns of opioid and cannabis use, (2) a concept analysis to delineate the concept of CPM, and (3) developing data-driven model cases for CPM. Study 1 was a cross-sectional survey with 110 AA adults (age 50 and older) in Louisiana, and study 2 was observational with 72 AA adults (age 55 and older) in Florida with self-reported osteoarthritis. Both samples were primarily women, 79-81% respectively. Only 33% and 1.3% reported using opioid analgesics, while less than 4% in both studies reported using medical or recreational cannabis. Using Walker and Avant’s concept analysis approach, the (A) attributes of CPM include lower side effect profile and social acceptability; (B) antecedents include chronic pain, motivation to seek alternative treatment, access to cannabis dispensaries, family and professional support, and health literacy; and (C) the consequences are pain relief, lower mortality, self-efficacy, reduced anxiety, and empowerment. Lastly, based on our quantitative and qualitative data, we developed two model cases demonstrating support for and against CPM. These results illuminate the need to further understand the barriers and facilitators to using opioids and CPM in older AAs.

